# Effects of selective EP2 receptor agonist, omidenepag, on trabecular meshwork cells, Schlemm’s canal endothelial cells and ciliary muscle contraction

**DOI:** 10.1038/s41598-021-95768-z

**Published:** 2021-08-10

**Authors:** Natsuko Nakamura, Megumi Honjo, Reiko Yamagishi, Nozomi Igarashi, Rei Sakata, Makoto Aihara

**Affiliations:** 1 Department of Ophthalmology, Graduate School of Medicine, The University of Tokyo, 7-3-1 Hongo Bunkyo-ku, Tokyo, 1138655 Japan; 2 Division of Vision Research, National Institute of Sensory Organs, National Hospital Organization Tokyo Medical Center, Tokyo, Japan

**Keywords:** Molecular medicine, Pathogenesis

## Abstract

This study investigated the effects of omidenepag (OMD), a novel selective EP2 receptor agonist, on human trabecular meshwork (HTM) cells, monkey Schlemm’s canal endothelial (SCE) cells, and porcine ciliary muscle (CM) to clarify the mechanism of intraocular pressure (IOP) reduction involving conventional outflow pathway. In HTM and SCE cells, the effects of OMD on transforming growth factor-β2 (TGF-β2)-induced changes were examined. The expression of actin cytoskeleton and extracellular matrix (ECM) proteins, myosin light chain (MLC) phosphorylation in HTM cells were evaluated using real-time quantitative PCR, immunocytochemistry, and western blotting. The expression of barrier-related proteins, ZO-1 and β-catenin, and permeability of SCE cells were evaluated using immunocytochemistry and transendothelial electrical resistance. The CM contraction was determined by contractibility assay. OMD significantly inhibited expression of TGF-β2 induced mRNA, protein, and MLC-phosphorylation on cytoskeletal and ECM remodeling in the HTM dose dependently. In SCE cells, OMD suppressed TGF-β2-induced expression of the barrier-related proteins and decreased SCE monolayer permeability. OMD at 3 µM significantly inhibited CM contraction, however, the effect was not significant at lower concentrations. IOP lowering effect of OMD through conventional outflow pathway is exerted by increasing outflow facilities with the modulation of TM cell fibrosis and SCE cell permeability.

## Introduction

Glaucoma is a progressive optic neuropathy characterized by the selective retinal ganglion cell death, and elevated intraocular pressure (IOP) is the most critical risk factor in all glaucoma subtypes^1,2^. IOP is defined by the balance between the production and outflow of aqueous humor (AH). There are two types of outflow routes: the conventional pathway and the uveoscleral pathway^3^. The main cause of elevated IOP in primary open angle glaucoma (POAG), the most common glaucoma subtype, is increased outflow resistance in the conventional outflow pathway^4^. Especially, cell cytoskeletal changes, a high-rigidity trabecular meshwork (TM), remodeling, excess deposition of extracellular matrix (ECM) proteins including collagen and fibronectin (FN), and increased intercellular adhesion between Schlemm's canal endothelium (SCE) cells are observed in glaucomatous eyes and are related to outlowresistance^5–8^. Previous studies have shown that high concentrations of biomarkers in the AH of POAG eyes such as transforming growth factor-β2 (TGF-β2) contribute to IOP elevation by promoting fibrosis and remodeling of the ECM^9,10^.

IOP reduction is currently the only reliable, evidence-based treatment of glaucoma, with pharmacological agents used as the first-line of therapy in most types of glaucoma, including prostaglandin (PG) analogs. PG is a bioactive lipid. There are four major PG types with biological functions: PGD2, PGE2, PGF2α and PGI2. Among them, PGF2α analogue, F prostanoid (FP) receptor agonist, is currently the first choice therapy for POAG^11,12^. PGF2α acts mainly on the longitudinal fibers of the ciliary muscle (CM) and increases the intracellular concentration of Ca^2+^ as a second messenger; the increased release of matrix metalloproteinases reduces the ECM in CM and promotes drainage from the uveoscleral outflow pathway^13–18^. However, even with the use of the PGF2α analogue, there is the possibility of insufficient IOP reduction or concerns regarding adverse reactions such as PG-associated periorbitopathy^12,19^. Consequently, there is a great clinical need for a first line drug treatments with potent IOP lowering effects that do not cause the adverse reactions associated with thePGF2α analogue.

Omidenepag isopropyl (OMDI) is a novel selective EP2 receptor agonist developed as an ophthalmic solution developed for the treatment of glaucoma and ocular hypertension in Japan^20–23^. There are four subtypes of G protein-coupled receptors that are ligands for PGE2: EP1, EP2, EP3 and EP4; the EP2 receptor, in particular, is widely expressed in the human eye including the TM, SCE and CM^24–26^. OMDI is converted to the active form of OMD during corneal penetration, and OMD increases intracellular levels of cyclic AMP as a second messenger^20^. In a previous study, OMD reportedly increased outflow facility through not only uveoscleral outflow but also conventional outflow in monkey eyes^27^. However, the detailed action of OMD, especially in tissues in conventional outflow, remains still unclear.

In this study, we evaluated the effects of OMD on human TM (HTM) cells, monkey SCE cells and porcine CM to investigate the IOP lowering mechanism of OMD in conventional outflow.

## Results

### Effects of OMD on TGF-β2-induced fibrogenic messenger RNA (mRNA) expression in HTM cells

**Figure 1:**
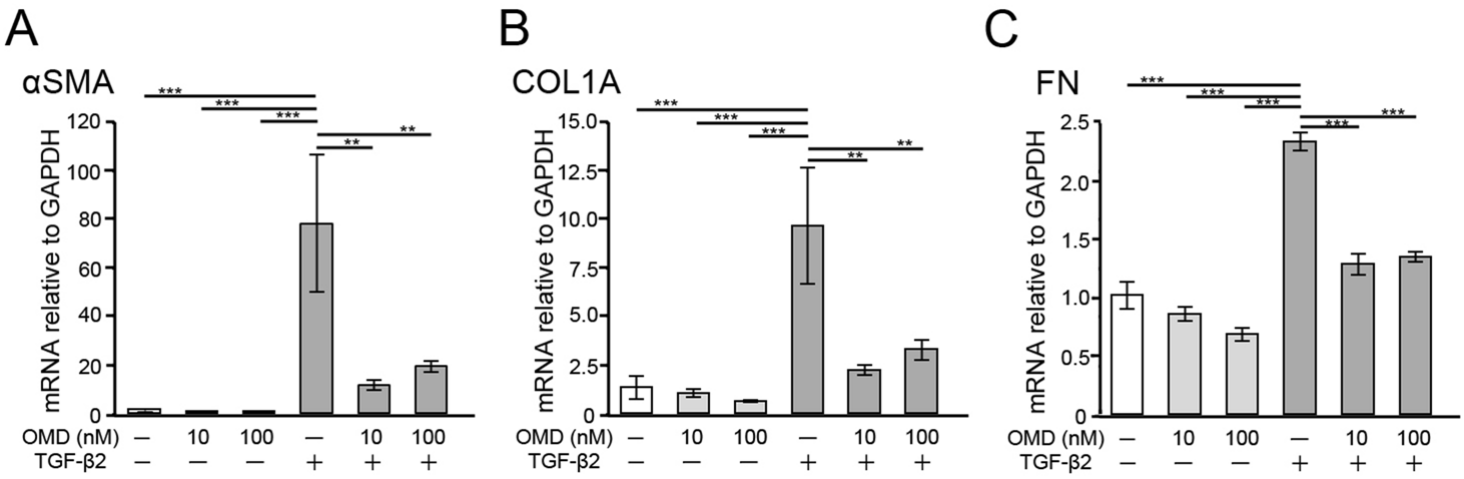
Effects of omidenepag (OMD) on transforming growth factor-β2-(TGF-β2)-induced mRNA expression of alpha smooth muscle actin (αSMA), collagen type I alpha 1 chain (COL1A), and fibronectin (FN) in human trabecular meshwork (HTM) cells. The mRNA expression of αSMA, COL1A, and FN in HTM cells after 24 h was evaluated using quantitative real-time polymerase chain reaction. TGF-β2-induced significant upregulation of αSMA, COL1A, and FN expression and OMD at 10 and 100 nM inhibited all changes significantly. Data are presented as the mean ± standard error (SE), *n* = 4. *P < 0.05, **P < 0.01, and ***P < 0.001 relative to the TGF-β2-treated group (Dunnett’s multiple comparisons test).

We investigated the effects of OMD on TGF-β2-induced fibrogenic changes in HTM cells. The mRNA expression of alpha-smooth muscle actin (αSMA), collagen type I, alpha 1 chain (COL1A), and FN was quantified using Quantitative Real-time Polymerase Chain Reaction (qRT-PCR) (Fig. 1). TGF-β2 significantly upregulated the mRNA expression of αSMA, COL1A1 and FN at 24 h (all, *P* < 0.001). OMD at both 10 and 100 nM significantly inhibited TGF-β2-induced mRNA upregulation (αSMA and COL1A, *P* < 0.01, FN, *P* < 0.001).

### Effects of OMD on TGF-β2-induced cytoskeletal and fibrotic protein expression in HTM cells

**Figure 2:**
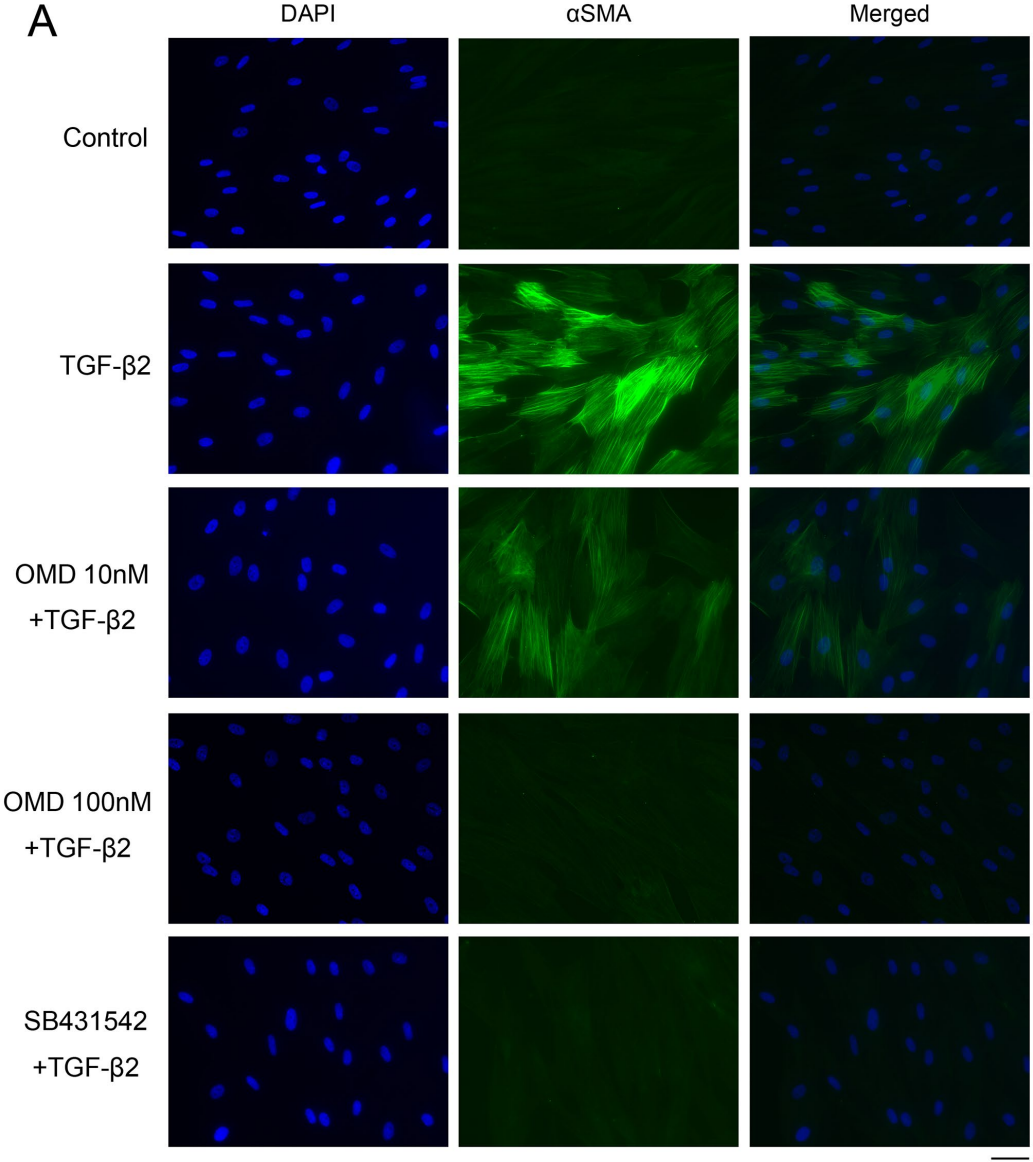

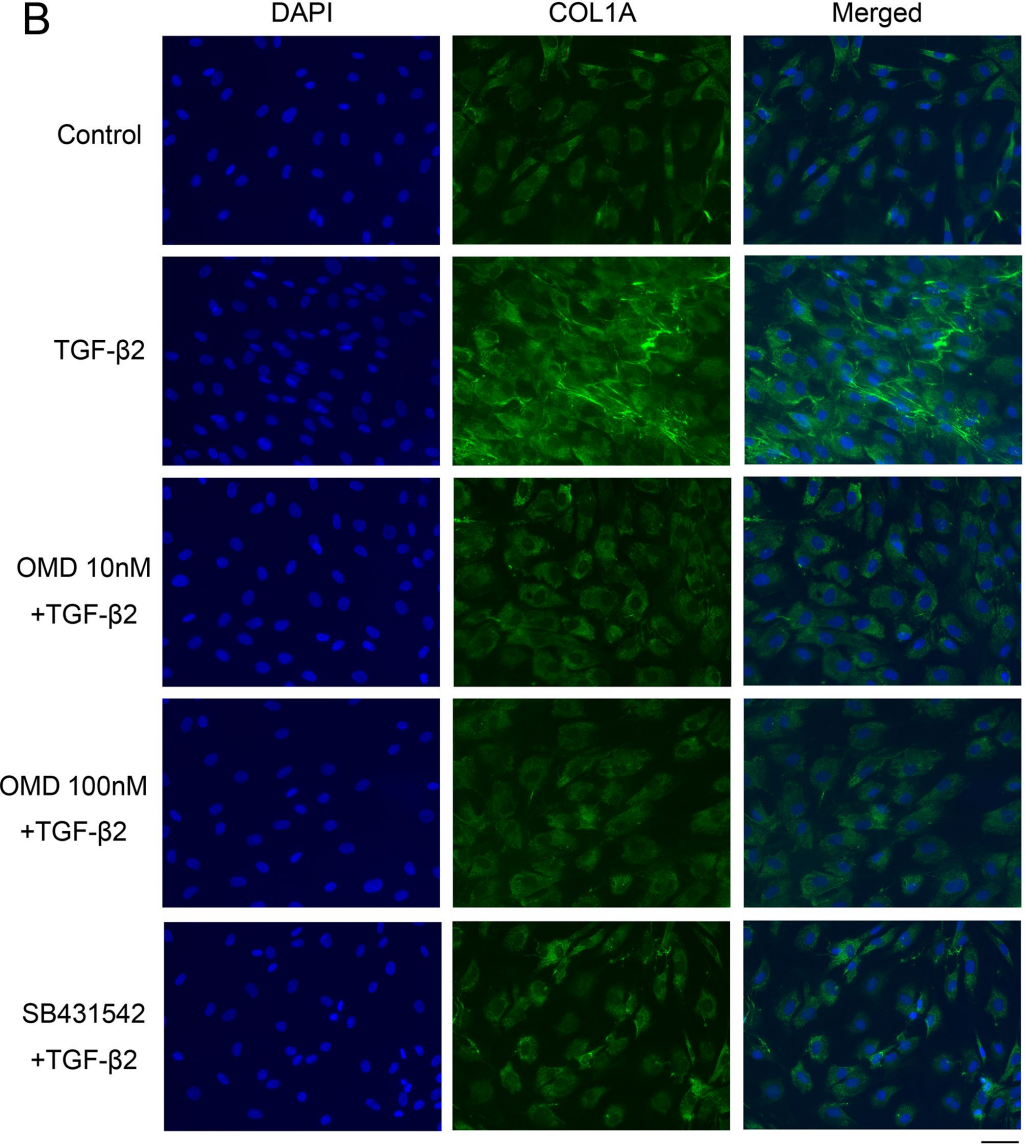

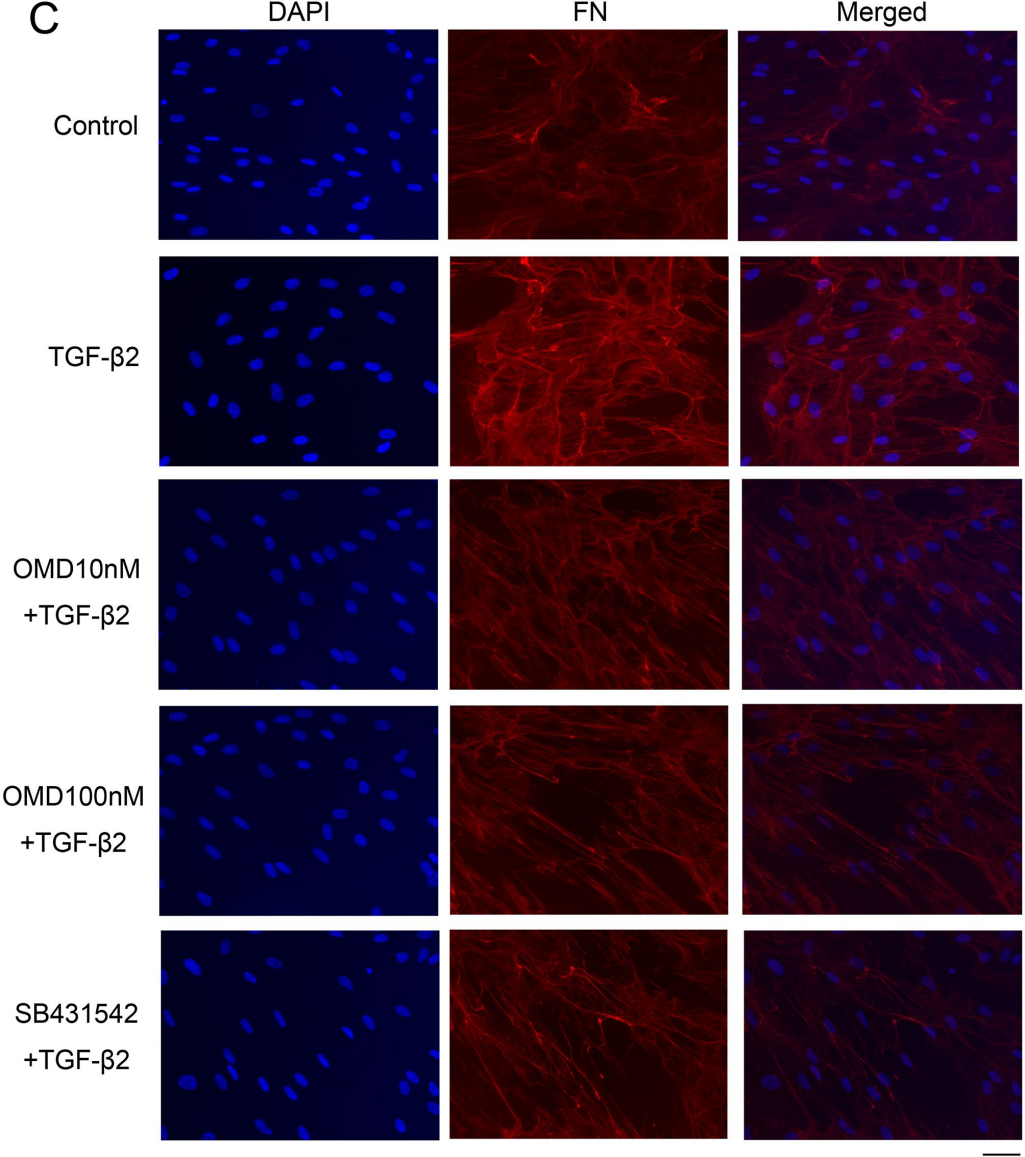

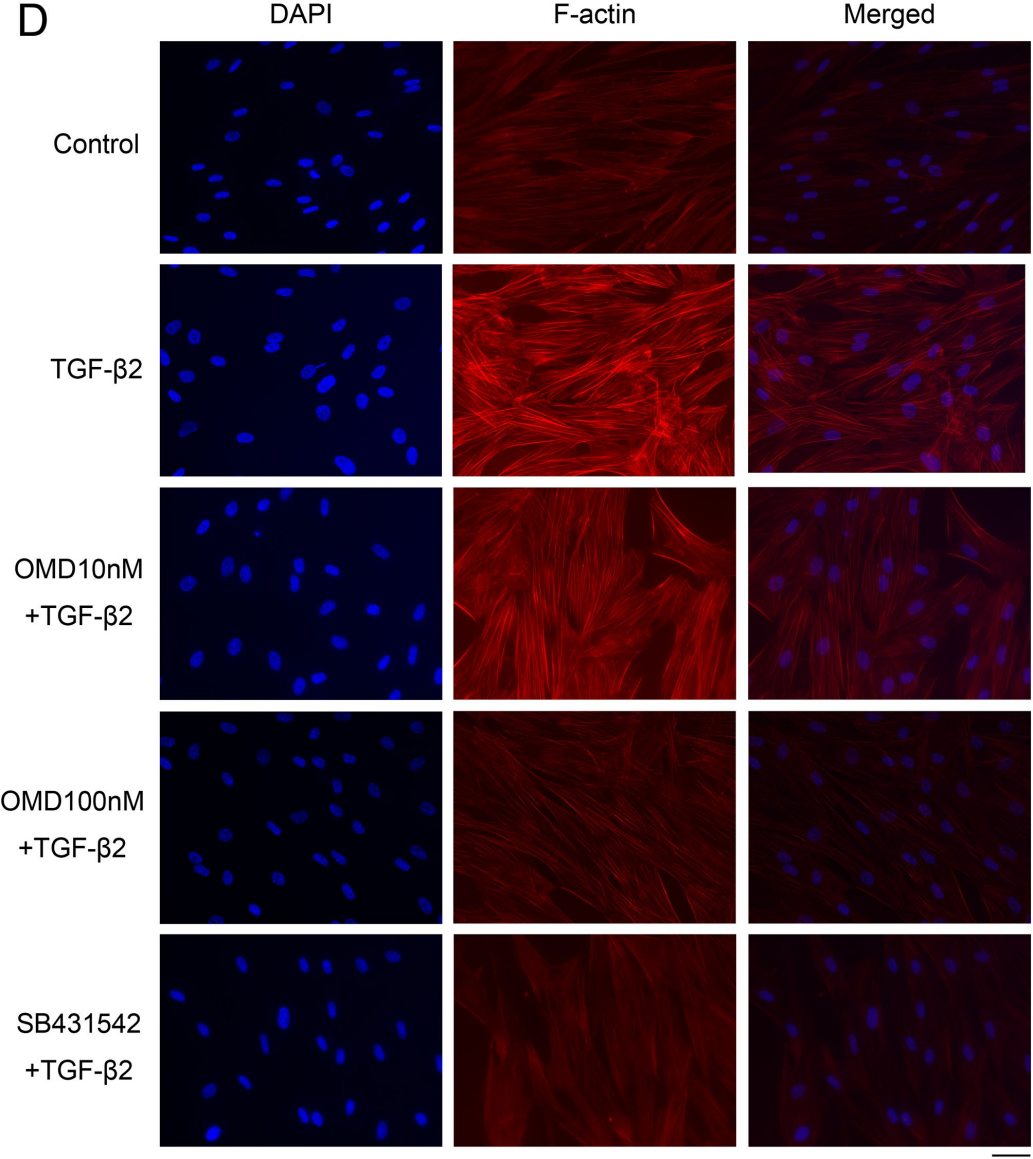
Effects of OMD on cytoskeletal and fibrotic protein expression in HTM cells. The effects of OMD on TGF-β2-induced cytoskeletal and fibrotic changes in HTM cells at 24 h were evaluated using immunocytochemistry. The samples were pretreated with TGF-β2 for 72 h and then treated simultaneously with TGF-β2 and OMD and fixed at 24 h after the stimulation with OMD. The left panels show cell nuclei counterstained with 4′,6-diamidino-2-phenylindole (DAPI; blue). The middle panels show cells stained for αSMA (green, **A**), COL1A (green, **B**), FN (red, **C**), and F-actin (red, **D**). The right panels show merged images. TGF-β2 significantly induced cytoskeletal and fibrotic changes in HTM cells and these changes were significantly suppressed by treatment with 100 nM OMD. Scale bars: 50 μm.

**Figure 3:**
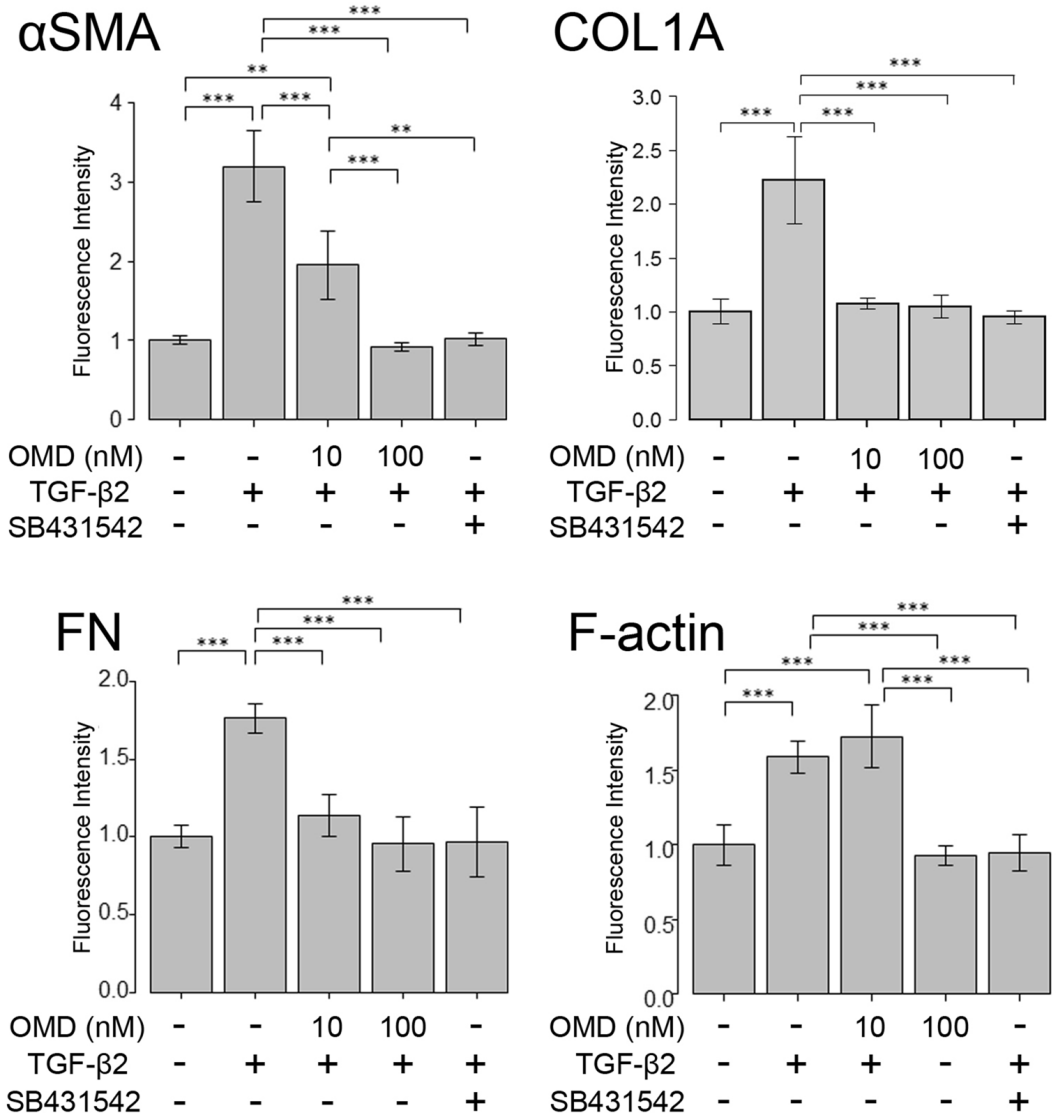
Quantitative results based on immunocytochemistry. Four images of each experiments were taken, and the fluorescence intensities were quantified. Data were presented as the mean ± standard deviation. **P < 0.01, ***P < 0.001.

Next, we assessed cytoskeletal and fibrotic changes in HTM cells using immunocytochemistry (Fig. 2). Pretreatment with TGF-β2 for 72 h significantly increased the expression of fibrotic proteins, αSMA (Fig. 2A), COL1A (Fig. 2B), and FN (Fig. 2C) and induced cytoskeletal changes as observed in actin bundles with F-actin staining after treatment with OMD for 24 h (Fig. 2D). The TGF-β2-induced changes were significantly suppressed by treatment with 100 nM OMD; however, the effect of 10 nM OMD was not significant. Similar levels of suppression were confirmed in 5 µM SB431542 (TGF-β type I receptor [TGFβRI]/activin receptor-like kinase 5 [ALK-5] inhibitor). The quantified intensities showed that αSMA, COL1A, FN and F-actin were upregulated with TGF-β2 stimulation, and the changes were attenuated with OMD at 10 and 100 nM and SB431542 (Fig. 3).

### Effects of OMD on tight-junction-related protein expression in SCE cells

**Figure 4:**
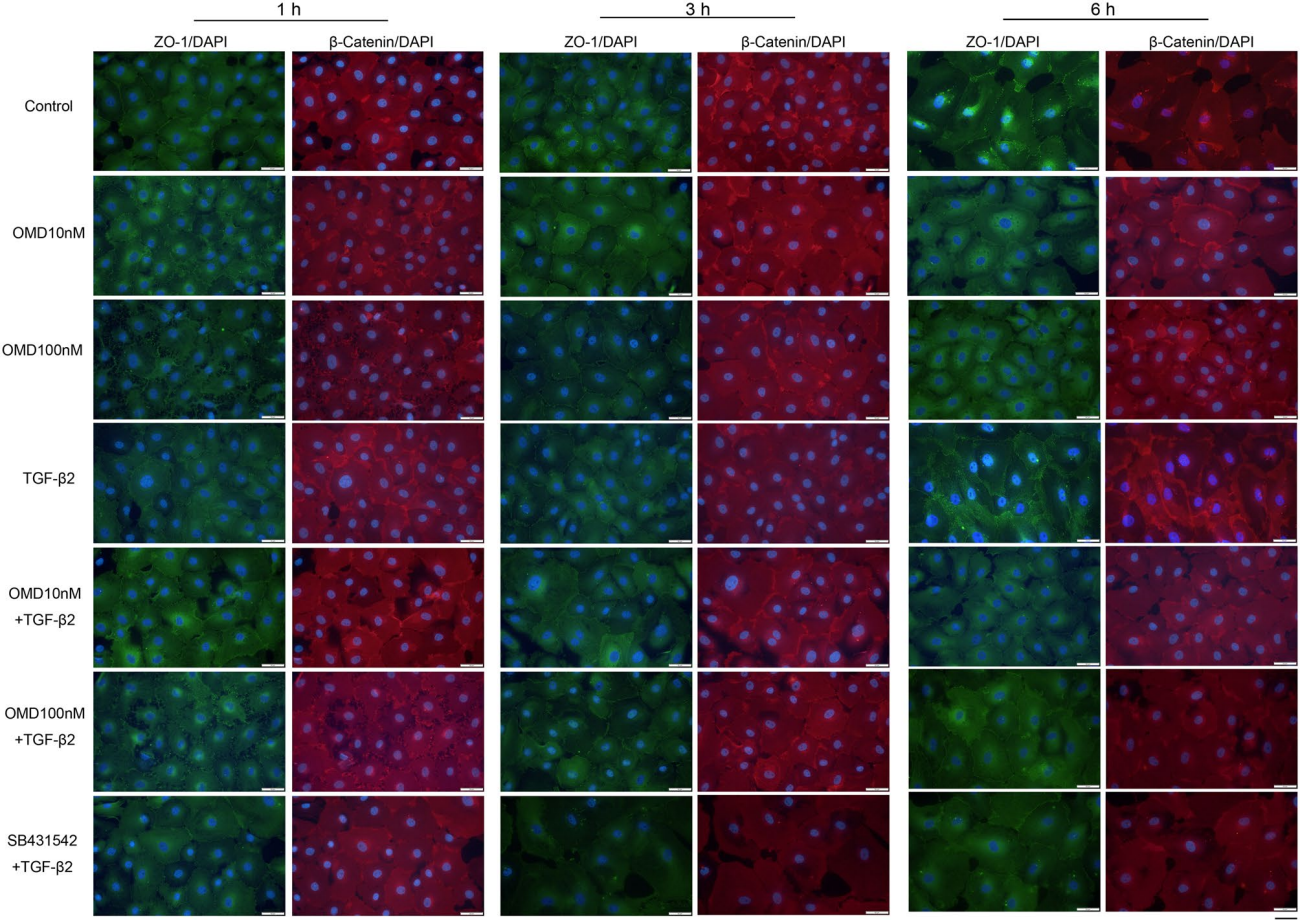
Effects of OMD on tight-junction-related protein expression in Schlemm’s canal endothelial (SCE) cells. The effects of OMD on TGF-β2-induced tight-junction-related protein expression in monkey SCE cells. The samples were pretreated with TGF-β2 for 1 h and fixed at 1, 3, 6 h after the stimulation with OMD. At 1 and 3 h after stimulation, SCE cells were stained with ZO-1 (green) and β-catenin (red). Cell nuclei were counterstained with DAPI (blue). Compared to the control, OMD (especially 100 nM) disrupted the stained areas of ZO-1 and β-catenin. Similar changes were seen between the OMD and TGF-β2 treatments. Scale bars: 50 μm.

In monkey SCE cells, we evaluated tight-junction-related protein expression at cell junctions (Fig. 4). When SCE cells were treated with OMD without any stimulation, no significant differences were observed between the control and OMD treatment. TGF-β2 stimulation increased the expression of ZO-1 and β-catenin at 1 h (TGF-β2 stimulation for 2 h in total including pretreatment). Following addition of OMD at 10 and 100 nM to samples pretreated with TGF-β2, the staining intensity of ZO-1 and β-catenin was reduced with OMD at 100 nM. The changes at the tight junction and adherence junction in response to 100 nM OMD continued at 3 h, and recovery to the previous states ultimately occurred at 6 h. Similar suppression of TGF-β2 induced effects was confirmed in the 5 μM SB431542 treatment.

### Effect of OMD on TGF-β2-induced MLC phosphorylation in HTM cells

**Figure 5:**
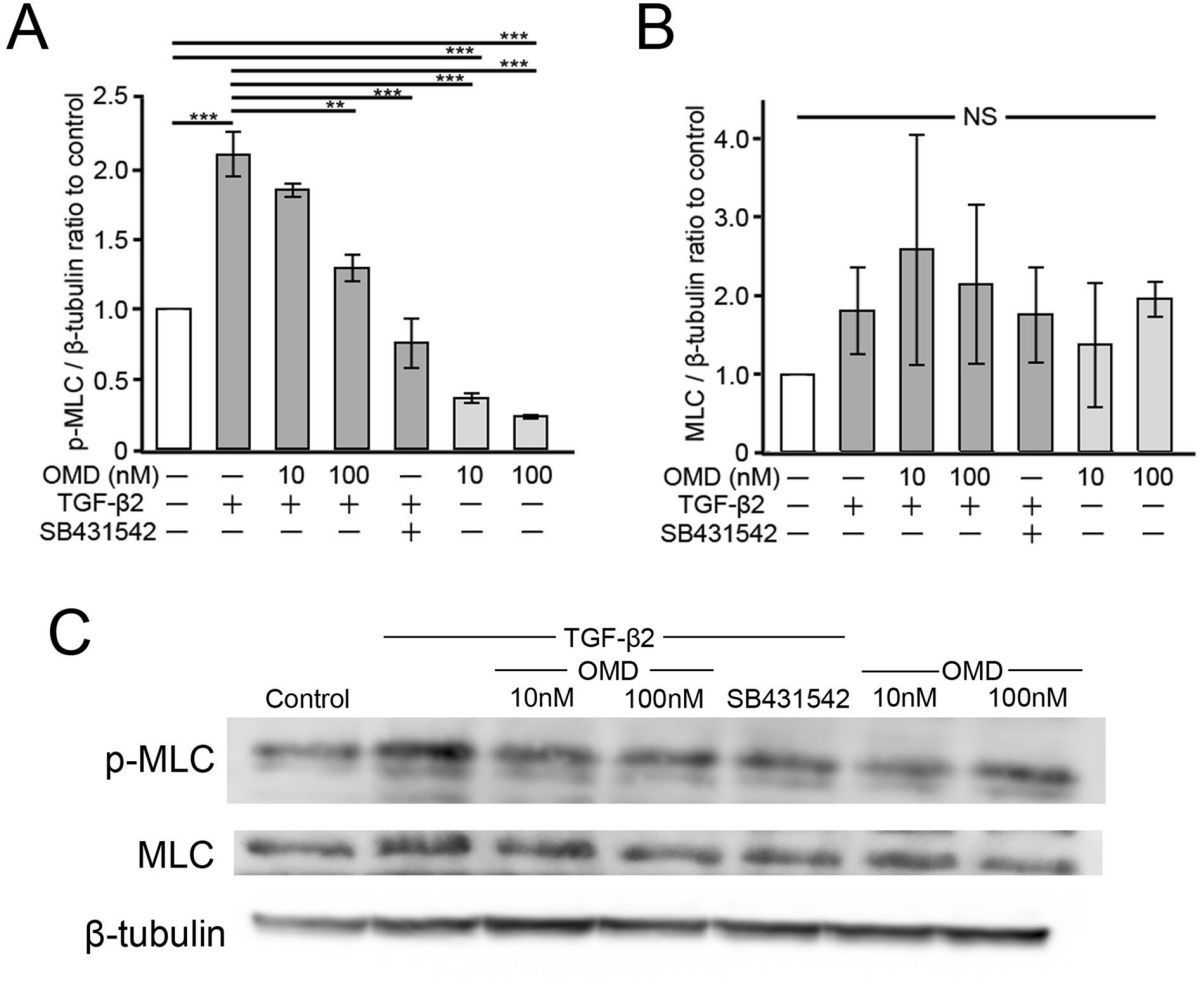
Effects of OMD on TGF-β2-induced myosin light chain (MLC) phosphorylation in HTM cells. HTM cells were treated with OMD (10, or 100 nM or 5 μM SB431542 and 5 ng/mL TGF-β2 or OMD alone for 24 h. The effects of OMD on the expression of phosphorylated MLC (p-MLC) and MLC are shown in (**A**) and (**B**), respectively. Representative Western blots of β-tubulin, p-MLC, and MLC are shown in (**C**). Data are presented as the mean ± SE, *n* = 3. *P < 0.05 and **P < 0.01 relative to control. ***P < 0.001 and **P < 0.01 relative to the TGF-β2-treated group (Dunnett’s multiple comparisons test).

To investigate the effect of OMD on TGF-β2-induced cytoskeletal changes, we performed Western blotting to detect the expression of phosphorylated myosin light chain (p-MLC) and myosin light chain (MLC). Phosphorylation of the MLC plays an important role in actomyosin contractility, and it has been reported that TGF-β2 induce its phosphorylation which increase in a time-dependent manner for at least 24 h^28–30^. After 24 h of stimulation, OMD at 10 and 100 nM significantly decreased the phosphorylation of MLC compared to the control group (Fig. 5A) (*P* < 0.05 and *P* < 0.01, respectively). TGF-β2 increased p-MLC significantly compared with the control group (*P* < 0.001), and OMD at 100 nM significantly suppressed this change (*P* < 0.01) (Fig. 5A). There were no significant differences in the expression of MLC (Fig. 5B). Similar suppression of TGF-β2 induced effects was confirmed in the 5 µM SB431542 treatment. Representative figures were shown in Fig. 5C.

### Effects of OMD on HTM-mediated collagen gel contraction

**Figure 6:**
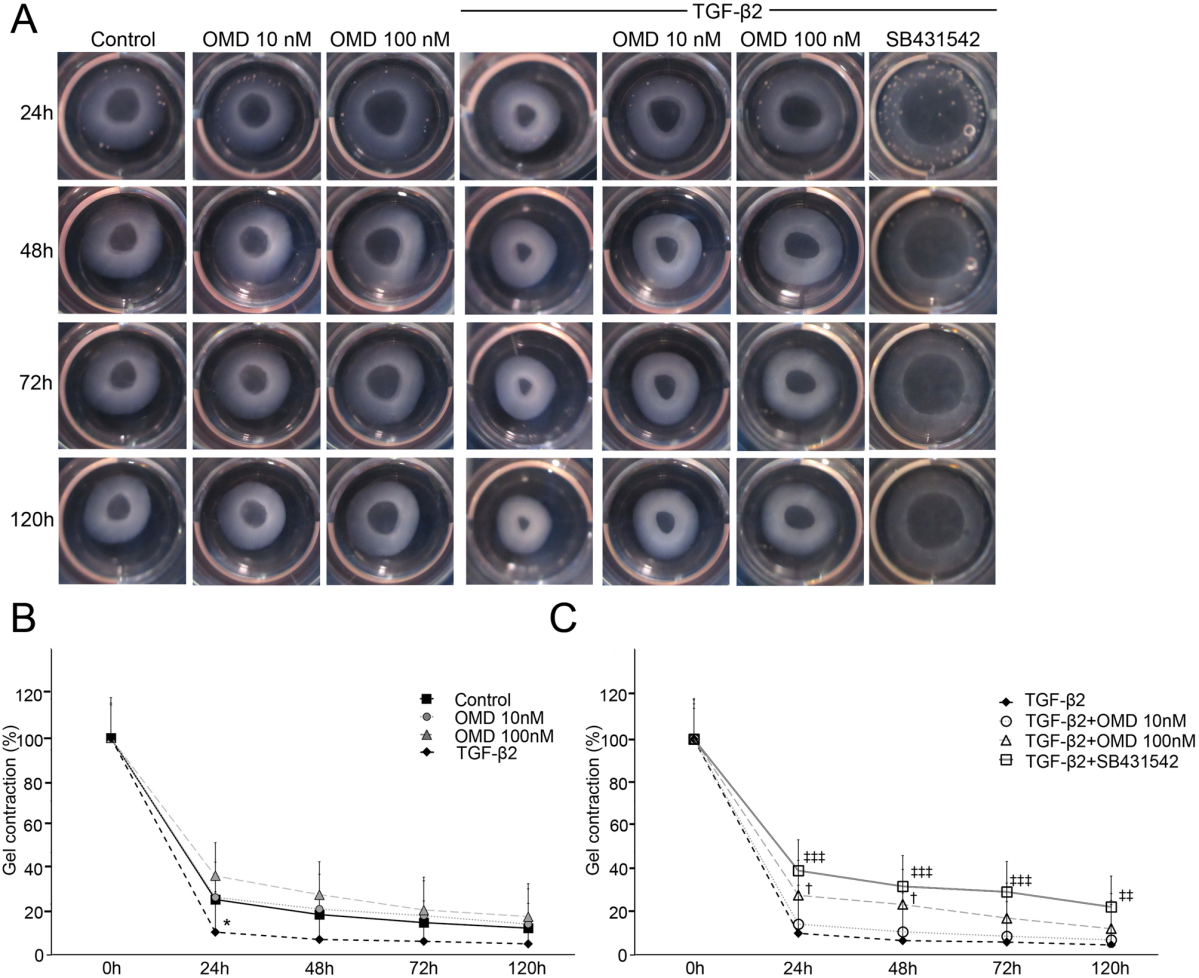
Effects of OMD on HTM-mediated collagen gel contraction. HTM-mediated contraction was evaluated using a collagen gel contraction assay. Collagen gels were incubated with TGF-β2 and OMD (10 or 100 nM) for 120 h. Representative photographs of the gels are shown in (**A**). Affected gel areas are expressed as the proportion of gel area that had decreased compared with the initial area of each group (**B**, **C**). Data are presented as the mean ± SE, *n* = 4. *P < 0.05 relative to control. ^†^*P* < 0.05 and ^‡^P < 0.001 relative to the TGF-β2-treated group (Dunnett’s multiple comparisons test).

We performed a collagen gel contraction assay to evaluate the effect of OMD on HTM-mediated gel contraction (Fig. 6A). When HTM cells were treated with OMD without any stimulation, OMD exhibited a tendency to suppress HTM contraction compared to the control; however, the change was not statistically significant (Fig. 6B). On the other hand, 100 nM OMD significantly inhibited TGF-β2-induced HTM contraction at 24 and 48 h (both, *P* < 0.05, respectively) (Fig. 6C). Similar significant suppression of on TGF-β2 induced effects was confirmed in the 5 μM SB431542 treatment at 24, 48, 72 and 120 h (all, *P* < 0.01) (Fig. 6C).

### Effects of OMD on transendothelial electrical resistance (TEER) in SCE cells

**Figure 7:**
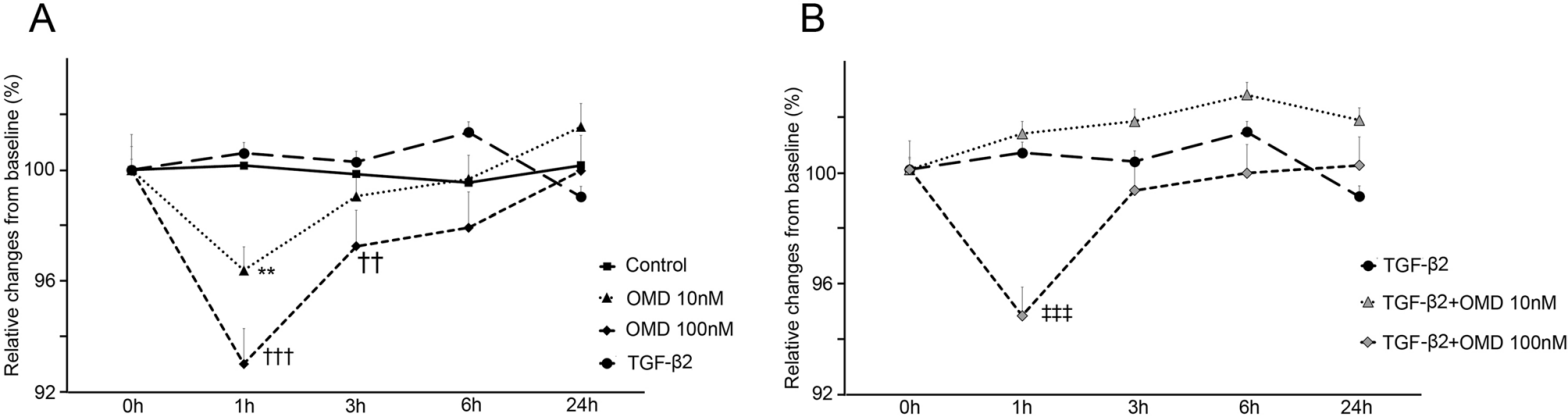
Effects of OMD on transendothelial electrical resistance (TEER) in SCE cells. The results of the TEER assay of the SCE cell monolayer are shown (**A**, **B**). At 1 h, 10 nM OMD decreased TEER significantly (**P < 0.01). OMD at 100 nM decreased TEER at 1 and 3 h compared to the control (^†††^*P* < 0.001 and ^††^*P* < 0.01, respectively) (**A**). OMD at 100 nM also decreased TEER at 1 h compared to TGF-β2 treatment (^‡‡‡^*P* < 0.001) (**B**). Data are shown as means ± SE, *n* = 4 (Dunnett’s multiple comparisons test).

We assessed TEER to investigate the cell–cell barrier function in monkey SCE cells. When SCE monolayers were treated with OMD without any stimulation, OMD at a concentration of 100 nM significantly reduced TEER at 1 h (*P* < 0.0001) (Fig. 7A). TGF-β2 did not result in any significant change in TEER compared with the control (Fig. 7A). At 1 h after OMD treatment with TGF-β2, OMD at a concentration of 100 nM led to significantly lower TEER compared with TGF-β2 alone (Fig. 7B).

### Effect of OMD on carbachol-induced contraction of CM

**Figure 8:**
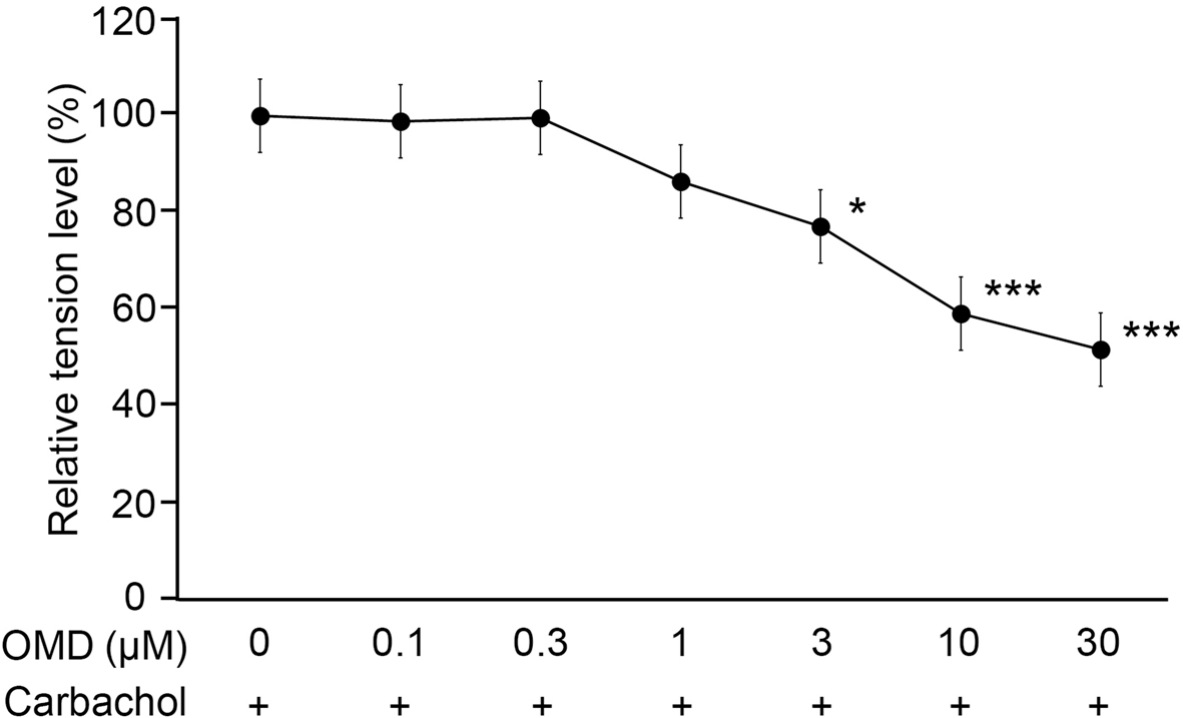
Effect of OMD on carbachol-induced contraction of ciliary muscle (CM). The effect of OMD on porcine CM contraction was measured using a force–length transducer. The relaxation responses of OMD were expressed relative to the maximum carbachol-induced contraction response. OMD inhibited CM contraction in a concentration-dependent manner. Data are presented as values relative to control values. Data are presented as the mean ± SE, *n* = 7. *P < 0.05 and ***P < 0.001 relative to baseline (Dunnett’s multiple comparisons test).

The level of carbachol-induced contraction was regarded as the baseline. OMD inhibited CM contraction in a concentration-dependent manner, and OMD at 3, 10, and 30 μM, significantly inhibited the carbachol-induced CM contraction (3 μM, *P* < 0.05; 10 and 30 μM, *P* < 0.001) (Fig. 8).

## Discussion

It has been suggested that the IOP-lowering effect of PG analogs, such as FP agonists, is induced by an enhancement of the uveoscleral outflow pathway^31^. Butaprost and another EP2 agonist, AH13205, induce CM relaxation and enlargement of spaces between CM bundles, which constitute a part of the uveoscleral outflow apparatus^14,32,33^. Therefore the enhancement of drainage via the uveoscleral outflow pathway related to ECM remodeling has been assumed to be a major mechanism underlying the IOP lowering effects of EP2 receptor agonists, as with in other PG analogs such as FP agonists. However, it is possible that the EP2 receptor agonists facilitate conventional outflow via the TM and Schlemm's canal, as the repeated dosing of butaprost reportedly leads to increased intercellular spaces and decreased collagen deposits not only in CM but also in the TM and Schlemm's canal tissues^20–23,34^. Indeed, Fuwa et al. reported that OMD lowered IOP in laser-induced ocular hypertensive monkey eyes by increasing both the outflow facility and uveoscleral outflow^27^. Enhancement of both outflow routes is beneficial in clinical practice and attributable to the strong IOP lowering effect of OMD. However, the precise mechanisms of EP2 receptor agonists, including OMD, or their effects on the conventional outflow pathway have not been explored previously.

In the present study, we revealed that OMD may regulate resistance in the conventional outflow pathway by increasing the outflow facility in association with the modulation of TM cell behavior and fibrosis as well as SCE cell permeability. Our results show that (1) OMD significantly inhibits TGF-β2 induced mRNA and protein expression involved in cytoskeletal rearrangement and ECM remodeling (Figs. 1, 2), (2) OMD suppresses MLC-phosphorylation and gel contraction in HTM (Figs. 5, 6), and (3) in SCE cells, OMD suppressed TGF-β2-induced expression of the barrier-related proteins, ZO-1 and β-catenin, and decreased SCE monolayer permeability as observed in the TEER assay (Fig. 7). In addition to the effects on cells in the conventional outflow pathway, OMD significantly inhibited carbachol-induced CM contraction at higher concentrations, which may contribute to an increase in uveoscleral outflow.

The conventional pathway through the TM and Schlemm’s canal is the major route of AH outflow regarding physiological conditions^35^, and AH outflow resistance is generated through the TM to Schlemm’s canal in both normal and glaucomatous eyes^1^. Cytoskeletal structural properties, adhesive interactions, SCE cell permeability, and various mediators in the AH are all potentially play important roles in outflow regulation^36^. Among the mediators, a high concentration of TGF-β2 in AH is a key factor in glaucoma progression, along with glaucomatous pathological changes in the outflow pathway such as fibrosis and deposition of ECM protein^37,38^. In the present study, we did not observe significant changes between the vehicle-treated control and OMD-treated groups of HTM and SCE cells. Therefore, we used TGF-β2 to investigate the mechanism of action of OMD mimicking the pathological condition of a conventional outflow pathway in POAG. As a result, we found that OMD significantly inhibited TGF-β2-induced cytoskeletal and fibrotic changes, gel contraction in HTM cells, and the barrier function of SCE cells. These results are in good agreement with those of previous studies reporting that butaprost, another EP2 receptor agonist, prevents TGF-β2–dependent collagen deposition, aSMA expression and MLC phosphorylation in HTM cells^30,39^. In addition, as stated above, topical instillation of butaprost in monkey eyes for 1 year reduced ECM deposition in the TM^40^. These results suggest that EP2 receptor agonists have long-lasting effects on HTM cells, including inhibiting ECM deposition and myofibrogenic changes. Moreover, butaprost or OMD is effective at lowering IOP both in ocular normotensive and ocular hypertensive animals; however, the IOP lowering effects are stronger and more likely in ocular hypertensive animals^20,40^. Kirihara et al. reported that 0.01% OMD significantly decreased the IOP in normotensive monkey eyes, by as much as 13.3 ± 1.2 mmHg from baseline levels at 6 h, whereas the maximal reduction achieved was 19.9 ± 3.0 mmHg in laser-induced ocular hypertensive monkeys^20^. It has been reported that the aqueous outflow through the conventional pathway becomes larger at higher IOPs; thus, the larger IOP reduction could be expected with the agent which increases conventional outflow, such as butaprost or OMD, in eyes with higher IOP^35^. In addition, given that OMD significantly interfered with the TGF-β2-induced increase in outflow resistance, it is reasonable to speculate that OMD may be effective at lowering IOP in TGF-β2 mediated pathological conditions associated with higher IOP in POAG.

SCE cells are the key sites responsible for AH outflow resistance in the conventional pathway with a barrier function maintained by tight junctions. ZO-1 and β-catenin are constituent proteins of the tight junction and adherent junction^41,42^. In the present study, TGF-β2 increased the immunostaining of ZO-1 and β-catenin and decreased SCE permeability; moreover, OMD at a concentration of 100 nM inhibited the changes. Interestingly, the effects of OMD on the SCE cell barrier and permeability were observed even without TGF-β2 stimulation, as shown in Fig. 7A. OMD significantly decreased TEER compared with the vehicle-treated control, whereas OMD did not exert significant effects without TGF-β2 stimulation in HTM cells. In other words, OMD affects the SCE cell barrier and permeability in a physiological manner, which may lead to IOP lowering in normotensive eyes. Wang et al. reported that butaprost predominantly affects SCE cells, as opposed to TM cells, in the conventional outflow pathway, which is in good accordance with the present study^43^. It has been reported that the IOP of normotensive monkeys treated with OMD was lowered in some cases to below 5 mmHg, the minimum level of IOP limited by the existence of episcleral venous pressure^20^. The predominant effect of OMD on SCE cells may explain such a strong effect on IOP. In the present study, we have found the changes at the tight junction and adherence junction in response to 100 nM OMD, which continued till 3 h, and recovery to the previous states ultimately occurred at 6 h. It is possible that there may exist a clustering of protein at adherence junctions or may effect on protein turnover.

Contraction and relaxation of the CM play an important role in the regulation of both conventional and unconventional outflows. It has been suggested that EP2 receptors are more abundant in CM than in TM and SCE cells^26^. Fujimoto et al. reported that 11-deoxy-16, 16-dimethyl PGE2 (100 nM), an EP2 agonist, suppresses carbachol-induced CM contraction^44^. Similar effects of two EP2 agonists, AH13205 and butaprost, on CM contraction have been reported^32,33^. In the present study, OMD also significantly inhibited carbachol-induced CM contraction at higher concentrations. Although it has been considered that the balance between the TM tissue and CM contractility antagonistically regulate total outflow, it has also been suggested that CM relaxation itself contributes little to IOP reduction in the conventional and unconventional pathways. ECM remodeling between CM longitudinal muscles has a greater effect on outflow regulation through the unconventional pathway^3^. In this study as well, the effective OMD concentration for CM contraction was 3 μM or more, which is higher than the tissue concentration by single eye drop, and it is unlikely that CM contraction itself affects the decrease in IOP. However, the significant effects of OMD on SCE permeability compared to the vehicle-treated control and CM contraction could be synergically attributable to the IOP lowering effects of OMD in ocular normotensive animals or humans. In addition, as repeated administration of butaprost reportedly decreased ECM deposition in CM bundles^40^, it is possible that OMD will have a similar effect. Further research is needed on the effects of OMD on CM and the contractile balance between the TM and CM.

There were several limitations in this study. We examined only the short-term responses to OMD in cultured cells of the conventional outflow pathway. Generally, remodeling of the ECM requires longer periods of time; thus, a longer evaluation period of the effects in conventional outflow tissue is required. Concomitantly, ex vivo or in vivo experiments are needed to analyze the actual response of conventional pathway tissues to OMD treatment, given that the TM, Schlemm’s canal and CM do not act individually but interact with one another. The relative contributions of uveoscleral flow and conventional outflow could also be involved in the lowering of IOP by OMD. Additionally, antagonistic TM–CM contractions should be investigated in vivo. In the present study, in vitro studies allowed us to observe the cellular functions of conventional outflow tissues. In future work, optical coherence tomography may provide insight into the in-vivo interactions between the TM, SC, and CM. In addition, in the present study, we used TGF-β2 as a stimulation to induce glaucomatous change in HTM and SCE cells, but whether the EP2 receptor pathway and TGF-β pathway interact or intersect is not demonstrated. Similarly, it has been reported that TGF-β2 induces cross-linked actin networks (CLAN) formation in TM cells^45^, but the effect of OMD on CLAN formation is not evaluated in this study. This should be studied in the future study. Finally, it is possible that OMD affects on clustering of protein at adherence junctions or affects on protein turnover. Also, there may be differences in the cellular localization of ZO-1 and/or β-catenin. In addition, it is necessary to evaluate the details of the junction formation using electron microscopy. So, further studies in future to investigate the effects of OMD on the barrier-related proteins are needed.

In conclusion, we demonstrated for the first time that the novel selective EP2 agonist, OMD, exerts significant effects on the conventional outflow pathway by modulating TM cell behavior and fibrosis as well as SCE cell permeability. OMD also significantly inhibited CM contraction, similar to other PG analogs including FP agonists and other EP2 agonists. OMD is expected to have substantial utility in affecting both conventional and unconventional outflow pathways via IOP-lowering mechanisms and providing a greater variety of pharmacological treatment options for glaucoma.

## Materials and methods

### Cell culture and drug treatment

Primary HTM cells and primary monkey SCE cells were respectively isolated from human donor eyes and cynomolgus monkey eyes as described previously^46–50^. The eyes were obtained and managed in compliance with the Declaration of Helsinki. Human donor eyes were obtained from the Rocky Mountain Lions Eye Bank (Aurora, CO, USA). HTM cells were isolated from donor corneoscleral rims and characterized as described previously^51^. Only well-characterized normal HTM cells, in which Dexamethasone (Dex)-induced myocilin (MYOC) upregulation was confirmed with quantitative qRT-PCR from passages 3 through 5 were used in our studies (Supplementary Fig. 1). Furthermore, for the HTM cell characterization, Dex-induced MYOC upregulation and immunocytochemistry using antibodies against Aquaporin 1 (AQP-1), Collagen Type IV (COL4A1), Matrix Gla Protein (MGP), tissue inhibitor of metalloproteinase (TIMP)-3, vimentin, and desmin was also performed according to previous reports (Supplementary Fig. 1)^52,53^. For the SCE cell characterization, lack of MYOC induction on treatment with Dex was confirmed as described previously (Supplementary Fig. 1)^54^. The HTM cells from three donor eyes (46y.o., 52y.o., 55y.o., without glaucoma) were used in this study. All animals used in this study were treated in accordance with the ARVO Statement for the Use of Animals in Ophthalmic and Vision Research and the dictates of the Animal Use Committee of the University of Tokyo. This study was in compliance with the ARRIVE guidelines for the in-vivo studies carried out on animals. Monkey eyes were obtained from a commercial laboratory (Shin Nippon Biomedical Laboratories, Kagoshima, Japan). The cells were cultured in Dulbecco’s modified Eagle’s medium (Life Technologies, Carlsbad, CA, USA) supplemented with 10% fetal bovine serum and antibiotic antimycotic solution (100×) (Sigma-Aldrich, St. Louis, MO, USA) at 37 °C under 5% CO2. SCE cells were cultured in gelatin-coated dishes. SCE cells from passages 3 through 5 were used in the experiments.

OMD was provided by Santen Pharmaceutical (Osaka, Japan) and dissolved in acidified dimethyl sulfoxide (DMSO). The tissue distribution data after instillation of EYBELIS™ (Santen Pharmaceutical) in monkey eyes were used to estimate the OMD concentration in the TM and CM as 59.8 and 1.8 nM, respectively (unpublished data provided from Santen). Thus, we set the concentrations of OMD at 0, 10, and 100 nM in the TM and SCE experiments. Using a CM contraction assay, we evaluated the effects of OMD at 0.1- and 10-nM concentrations, which are within the estimated concentrations clinically, however, no significant effects were observed. Thus, we extended the concentration range from 100 nM to 30 μM to confirm the concentration-dependent effect.

All in-vitro experiments were performed after 24 h of serum starvation. When cells were stimulated with 10 ng/mL human recombinant TGF-β2 (Sigma-Aldrich), they were treated simultaneously with OMD and 5 μM SB431542 (Fujifilm, Osaka, Japan) unless otherwise stated. The same amount of DMSO used for dissolving OMD was added to the control group.

### RNA extraction and qRT-PCR with TM cells

HTM cells were cultured in 24-well plates until confluent. After 24 h of the stimulation with OMD and TGF-β2, the cells were lysed with TRIzol reagent (Life Technologie), and mRNA was isolated with chloroform and isopropyl alcohol. To characterize HTM cell strain profiles, the cells were treated with 100 nM or 500 nM Dex for 7 days without serum starvation. The isolated mRNA was processed to synthesize complementary DNA (cDNA) with a PrimeScript RT Reagent kit (Takara Bio, Shiga, Japan). The mRNA levels were quantified using qRT-PCR analysis of cDNA with SYBR Premix Ex Taq II (Tli RNase H Plus) and the Thermal Cycler Dice Real-Time System II (Takara Bio) with the DDCt method. The sequences of the PCR primers applied are as follows: glyceraldehyde 3-phosphate dehydrogenase (GAPDH): forward 5′-GAGTCAACGGATTTGGTCGT-3′ and reverse 5′-TTGATTTTGGAGGGATCTCG-3′; αSMA: forward 5′-CCGACCGAATGCAGAAGGA-3′ and reverse 5′-ACAGAGTATTTGCGCTCCGAA-3′; COL1A: forward 5′-CAGCCGCTTCACCTACAG-3′ and reverse 5′-TTTTGTATTCAATCACTGTCTTGCC 3′; FN: forward 5′-AAACCAATTCTTGGAGCAGG-3′ and reverse 5′-CCATAAAGGGCAACCAAGAG-3′; MYOC, forward 5′-TACACGGACATTGACTTGGC-3′ and reverse 5′-ATTGGCGACTGACTGCTTAC-3′.

The target gene expression levels were normalized to that of GAPDH and were presented relative to the control group. All tests were performed in triplicate to confirm reproducibility.

### Immunocytochemistry of TM cells and SCE cells

Immunocytochemical analysis of the HTM cells and monkey SCE cells was performed as described previously^55^. HTM cells were cultured in 24-well plates with glass coverslips until confluent. After serum starvation, the cells were pretreated with TGF-β2 (10 ng/mL) for 72 h before addition of OMD (10 or 100 nM) or SB431542. After 24 h of the stimulation with OMD and SB431542, simultaneously with TGF-β2, the cells were fixed with 4% paraformaldehyde for 15 min, permeabilized with 0.3% Triton X-100 in phosphate-buffered saline (PBS) for 10 min and blocked with Blocking One Histo (Nacalai Tesque, Kyoto, Japan) for 30 min at room temperature. The cells were incubated at 4 °C overnight with the following primary antibodies: anti- αSMA (1:500; DAKO, Tokyo, Japan), anti-collagen type 1 (1:100, Sigma-Aldrich), anti- FN (1:100; Sigma-Aldrich), anti-AQP-1 antibody (1:500; Santa Cruz Biotechnology, Inc., Santa Cruz, CA), anti-COL4A1 antibody (1:500; OriGene Technologies Inc., Rockville, MD, USA), anti-MGP antibody (1:500; Santa Cruz Biotechnology), anti- TIMP-3 antibody (KYOWA PHARMA CHEMICAL, CO., LTD, Toyama, Japan), anti-vimentin antibody (1:1000; Abcam, Cambridge, MA, USA), and anti-desmin antibody (1:200; Abcam). The cells were washed with PBS and incubated with the secondary antibody for 30 min at room temperature. The secondary antibody was Alexa Fluor 488 or 594 (1:1000; Thermo Fisher Scientific, Waltham, MA, USA). Phalloidin -rhodamine (7:1000, Sigma-Aldrich) was used for F-actin staining, Nuclei were stained with 4′,6-diamidine-2-phenylindole dihydrochloride solution (DAPI; 4 μg/mL; FUJIFILM Wako Pure Chemical, Osaka, Japan). Images were obtained with the fluorescence microscopy (model IX71; Olympus, Tokyo, Japan). The intensities were quantified using ImageJ software (http://imagej.nih.gov/ij/; provided in the public domain by the National Institutes of Health, Bethesda, MD, USA). SCE cells were cultured in 24-well plates with gelatin-coated glass coverslips. After serum starvation, the cells were pretreated with TGF-β2 (10 ng/mL) for 1 h before addition of OMD (10 or 100 nM) or SB431542. After 1, 3 and 6 h of the stimulation with OMD and SB431542, simultaneously with TGF-β2, the same procedure as that described for HTM cells was carried out. The primary antibodies for SCE cells were anti-ZO-1 (1:100; Abcam) and anti-β-catenin (1:100; Abcam, Cambridge, UK).

### Western blotting of TM cells

The expression of p-MLC was determined using Western blotting as described previously^46^. HTM cells were cultured in six-well plates until confluent. After 24 h of the stimulation with OMD, SB431542, simultaneously with TGF-β2, the cells were washed three times with PBS and lysed in radioimmunoprecipitation assay buffer (Thermo Fisher Scientific) on ice. The total protein concentrations of the cell lysates were quantified using a BCA Protein Assay kit (Thermo Fisher Scientific K.K., Kanagawa, Japan). The proteins were dissolved in sample buffer (Thermo Fisher Scientific K.K.) at 65 °C; the same amounts of protein samples were separated on 4–12% precast polyacrylamide gels (BIO-RAD Laboratories, Hercules, CA USA) via sodium dodecyl sulfate–polyacrylamide gel electrophoresis and transferred to polyvinylidene fluoride membranes (BIO-RAD Laboratories). The membranes were blocked with Blocking One-P (Nacalai Tesque) and incubated with rabbit polyclonal antibody against p-MLC (1:1000; Cell Signaling Technology, Danvers, MA, USA) in PBS. After the membranes were washed in Tris-buffered saline with Tween 20 (TBST), they were incubated with a horseradish peroxidase-conjugated secondary antibody (H goat anti-rabbit IgG, 1:2000, Thermo Fisher Scientific) in PBS. Afterwards, the membranes were washed in TBST and visualized using an enhanced chemiluminescence substrate (Thermo Fisher Scientific). Protein bands were detected by Image Quant LAS 4000 mini (GE Healthcare, Chicago, IL, USA). All membranes were stripped of antibodies solution in Western blot stripping (Nacalai Tesque) and blocked with Blocking One. The membranes were then incubated with mouse monoclonal MLC antibody (1:1000; Sigma-Aldrich) and β-tubulin (1:1000; FUJIFILM Wako Pure Chemical), and subsequently with H goat anti-mouse IgG, with β-tubulin used as the loading control. Densitometry of scanned films was performed with ImageJ 1.49 (http://imagej.nih.gov/ij/; National Institutes of Health, Bethesda, MD, USA); the results are expressed relative to the loading control (β-tubulin).

### Collagen gel contraction assay with TM cells

Collagen gel contraction in HTM cells was assessed using a Cell Contraction Assay kit (Nitta Gelatin, Inc., Osaka, Japan) as described previously^55^. Briefly, collagen gel was produced, as specified by the manufacturer, and placed in 24-well plates. HTM cells were cultured on the collagen gel until confluent. The tops of the collagen gel were stimulated, and the gels were freed from the walls of the culture wells. The gel areas were recorded photographically at 0, 24, 48, 72, 120 h and analyzed with ImageJ software (National Institutes of Health, Bethesda, MD, USA). The data analysis was in the masked manner. Changes in area for each group are shown as the gel contraction (%) compared with the gel at the start of the incubation time in bar graphs.

### TEER of SCE cells

TEER and the permeability of the SCE cell monolayer were assessed as described previously^47,48^. SCE cells were cultured in polycarbonate membrane inserts (pore size, 0.4 μm; diameter, 12 mm; Corning Transwell; Sigma-Aldrich) in 24-well plates for 2 weeks until confluent. The volume of the supplied medium was 1.2 mL in basal chambers and 200 μL in apical chambers of the transwells. After serum starvation, the SCE cells were stimulated with TGF-β2 (10 ng/mL). At 1 h after of the stimulation, OMD (10 or 100 nM) or SB431542 was applied to both basal and apical chambers. TEER was measured at 1, 3, 6, and 24 h after the stimulation with OMD and SB431542. Each experiment was repeated at least three times.

### Porcine CM contraction assay

CM contraction was assessed as described previously^29,56^. Briefly, fresh enucleated porcine eyes were obtained from Tokyo Shibaurazouki Co. Ltd (Tokyo, Japan), and CM strips were resected. The removed CM strips (approximately 2 mm × 5 mm) were placed in Krebs–Henseleit solution. The strips were fixed to an isometric transducer (Nihon Kohden Corp., Tokyo, Japan) preloaded with a 10-mL Magnus tube. Carbachol (10^−6^ M) was applied to confirm the contractile stability of the muscle. The strips were rinsed twice and equilibrated for 120 min. After responses to carbachol (10^−6^ M) were obtained repeatably, the strips were relaxed by adding OMD (0.1, 0.3, 1, 3, 10, and 30 µM). The mean isometric force measurement is presented as the value relative to that of the response at the maximum carbachol concentration. The relaxation responses to OMD were obtained in a cumulative manner in the CM strips. Relaxation responses are expressed as percentages of the maximum effect (100%) elicited by pilocarpine in each strip.

### Statistical analysis

Statistical analyses were performed with JMP Pro 14 software (SAS Institute Inc., Cary, NC, USA). All experimental data are presented as the mean ± SE. Paired *t*-test was used to compare two groups. Differences in data among groups were analyzed using one-way analysis of variance and Dunnett’s multiple comparisons test. A *P *value < 0.05 was considered to indicate statistical significance in all analyses.

## Supplementary Information

Supplementary Figure S1.

Supplementary Legend.
