# Supplementary material for: Effects of selective EP2 receptor agonist, omidenepag, on trabecular meshwork cells, Schlemm’s canal endothelial cells and ciliary muscle contraction
Source: Sci Rep. 2021 Aug 10;11:16257. doi: 10.1038/s41598-021-95768-z (PMC8355290; doi:10.1038/s41598-021-95768-z)

Effects of a selective EP2 receptor agonist, omidenepag, on trabecular meshwork cells, Schlemm's canal endothelial cells and ciliary muscle contraction

Natsuko Nakamura[1,2] MD, Megumi Honjo[1*], MD, PhD, Reiko Yamagishi[1], MD, PhD,

Nozomi Igarashi[1*], MD, PhD, Rei Sakata[1*], MD, PhD, Makoto Aihara[1,] MD, PhD

1. Natsuko Nakamura

Email: ntk.nakamura.77.30@gmail.com

Institution: The University of Tokyo

Department: Department of Ophthalmology

Address: 7-3-1 Hongo, Bunkyo-ku, Tokyo, Japan

Zip code: 113-8655

Work phone: 81-3-3815-5411

2. Megumi Honjo

Email: honjomegumi@gmail.com

Institution: The University of Tokyo

Department: Department of Ophthalmology

Address: 7-3-1 Hongo, Bunkyo-ku, Tokyo, Japan

Zip code: 113-8655

Work phone: 81-3-3815-5411

3. Reiko Yamagishi

Email: yamagishi015@gmail.com

Institution: The University of Tokyo

Department: Department of Ophthalmology

Address: 7-3-1 Hongo, Bunkyo-ku, Tokyo, Japan

Zip code: 113-8655

Work phone: 81-3-3815-5411

4. Nozomi Igarashi

Email: kindenauthor@yahoo.co.jp

Institution: The University of Tokyo

Department: Department of Ophthalmology

Address: 7-3-1 Hongo, Bunkyo-ku, Tokyo, Japan

Zip code: 113-8655

Work phone: 81-3-3815-5411

5. Rei Sakata

Email: reisakata@gmail.com

Institution: The University of Tokyo

Department: Department of Ophthalmology

Address: 7-3-1 Hongo, Bunkyo-ku, Tokyo, Japan

Zip code: 113-8655

Work phone: 81-3-3815-5411

6. Makoto Aihara

Email: aihara-tky@umin.net

Institution: The University of Tokyo

Department: Department of Ophthalmology

Address: 7-3-1 Hongo, Bunkyo-ku, Tokyo, Japan

Zip code: 113-8655

Work phone: 81-3-3815-5411

Supplementary Figure 1

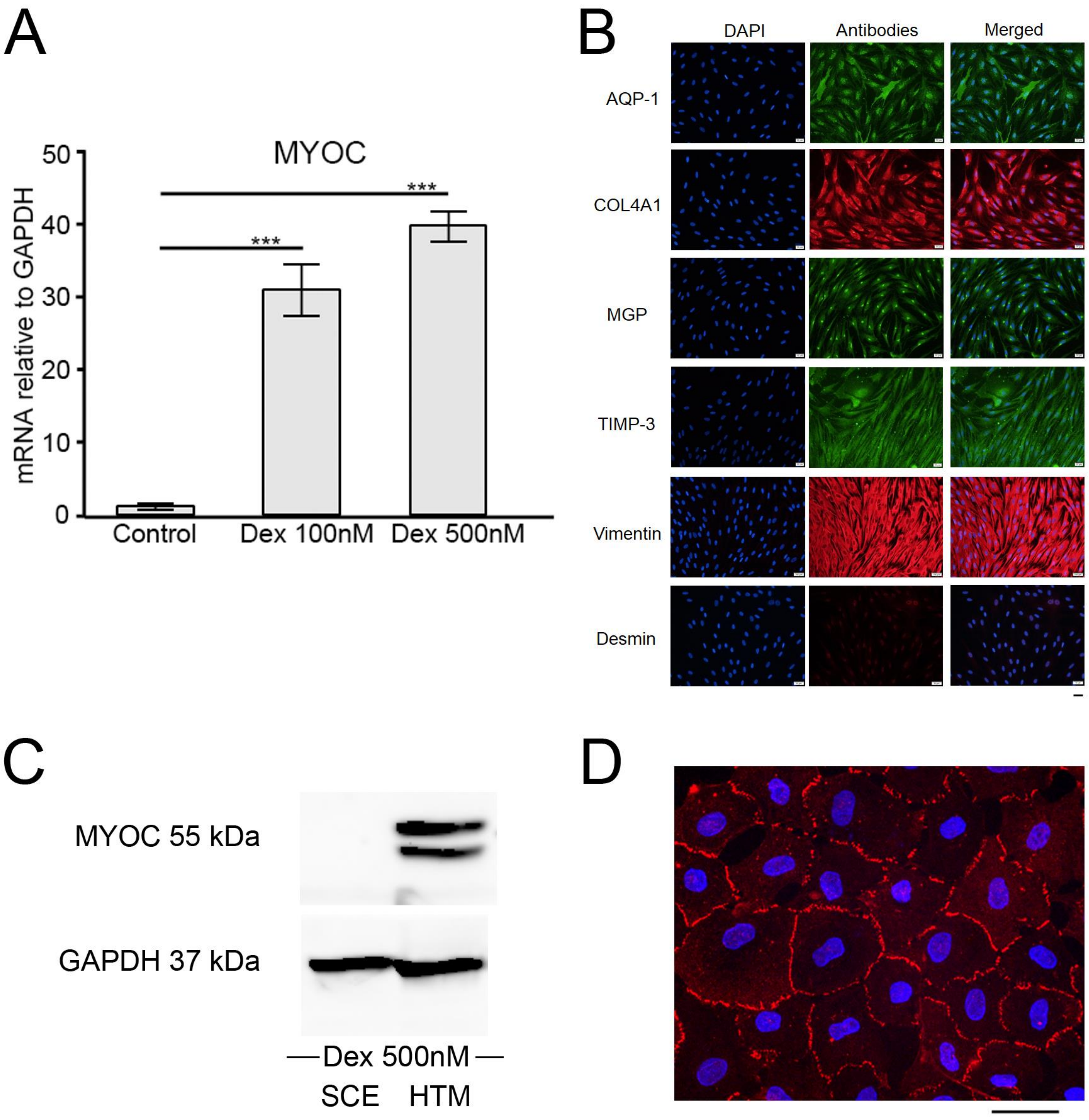

Supplementary Data

GAPDH

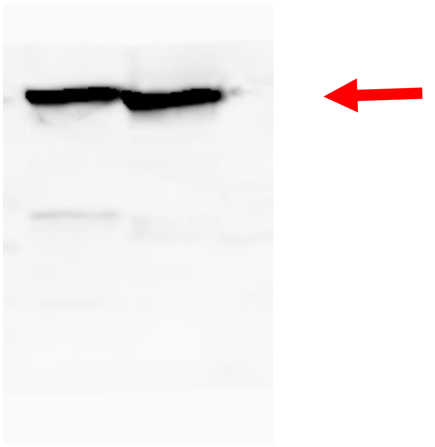

GAPDH:37kDa

SCE HTM

MYOC

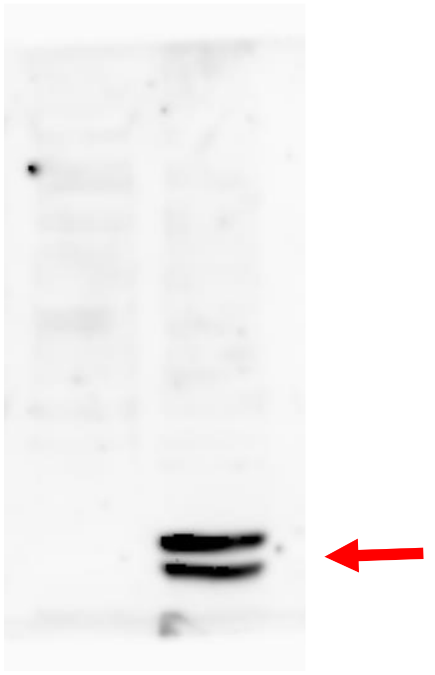

MYOC:55kDa

SCE HTM

Supplementary Data

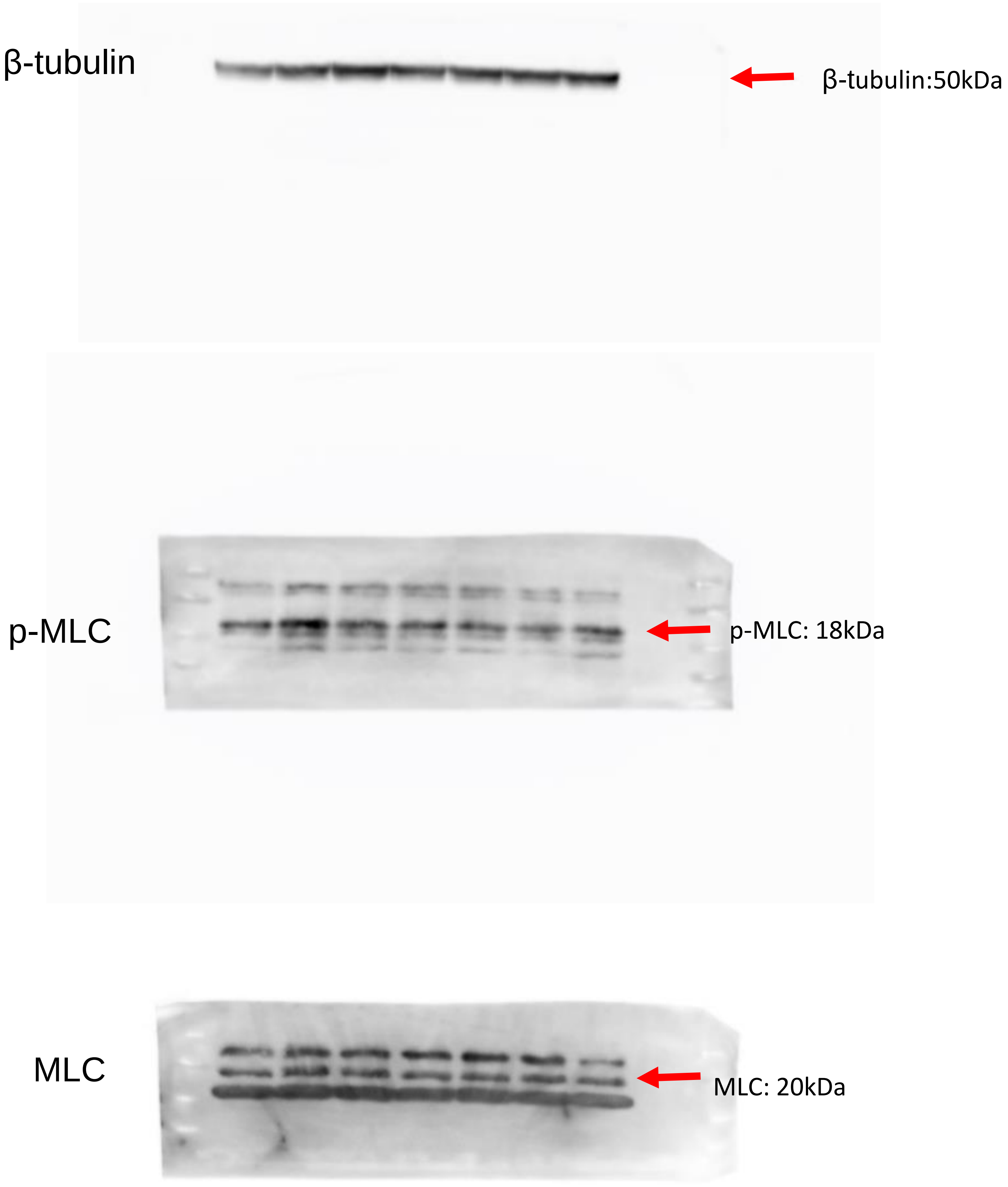

Supplement: Supplementary file 1 — Supplementary Figure S1. [file 41598_2021_95768_MOESM1_ESM.pdf]
