# Supplementary material for: Effects of selective EP2 receptor agonist, omidenepag, on trabecular meshwork cells, Schlemm’s canal endothelial cells and ciliary muscle contraction
Source: Sci Rep. 2021 Aug 10;11:16257. doi: 10.1038/s41598-021-95768-z (PMC8355290; doi:10.1038/s41598-021-95768-z)
Supplement: Supplementary file 2 — Supplementary Legend. [file 41598_2021_95768_MOESM2_ESM.pdf]

Supplemental Figure 1. Characterization of HTM cells and SCE cells. (A) The significantly increased mRNA expression of MYOC in HTM cells was confirmed by 100nM and 500 nM Dex treatment (7 days). ***$P < 0.001$. (B) The panels show cells stained for DAPI (blue), endothelial cell markers and mesenchymal markers, merged images from left to right. The HTM cells used in the study were positive for AQP-1, COL4A1, MGP, TIMP-3, vimentin and desmin, but the cells were negative for desmin. Bar, 200 µm. (C) Dex treatment (500 nM) for 3 days increased MYOC expression in HTM cells. On the other hand, there were no Dex-induced MYOC expression in SCE cells. (D) SCE cells were stained with ZO-1 (green), and cell nuclei were counterstained with DAPI (blue). Scale bar: 50 µm.
